# Death of Prof. James Hadley, M. D.—Meeting of the Members of the Medical Profession

**Published:** 1869-10

**Authors:** Julius F. Miner

**Affiliations:** Secretary


### Death of Prof. James Hadley, M. D.—Meeting of the Members of the Medical Profession.

At a meeting of the members of the medical profession, convened on account of the death of Prof. JAMES HADLEY, M. D., Prof. JAMES P. WHITE was called to the chair and remarked, as follows: I am most profoundly moved in making the announcement of the death of a venerable and most highly respected member of the profession, Prof. JAMES HADLEY, who died October 18th, at his residence in Buffalo, aged eighty-four years. Nearly forty years since I had the pleasure of listening to his able course of Lectures on Chemistry in the then flourishing college in Fairfield, N. Y., in connection with over two hundred fellow students, and from that period to the present have known him well, with daily increasing respect and veneration. Dr. HADLEY was also Professor of Chemistry in the Geneva Medical College, and always proved himself a most able, scientific, successful and popular teacher, from whom very many of the older members of the profession in the State of New York have learned Chemistry. In his favorite branch of medical knowledge he was unsurpassed in merit, yet without pretention, without show. He came to Buffalo later in life, but has never been idle in his department of the profession. A more blameless man I never knew; kind, generous, noble-hearted and true—under all circumstances incorruptible; an honor to his profession, to humanity and the world. It is fitting that we make note of the departure of a man of such excellence and purity, of such eminence and worth, and resolve to copy his example and imitate his virtues. He has departed in the full ripeness of mature age, and we are called upon to notice and profit by the close of a complete life.

Dr. ROCHESTER said that the remarks of Prof. WHITE were so full and appropriate, that there was little left for him to do but to corroborate them. He, too, had personal reminiscences of Prof. HADLEY extending over a period of five and twenty years, both when he was an academic and a medical student. Prof. HADLEY always secured the respect and esteem of his pupils to a very marked degree. He was confided in by them to such an extent that his arbitration, counsel and advice were usually sought for by them in their various troubles and perplexities; and it was always given in such a just, warm and cordial way, that he was generally spoken of as "Father" HADLEY—in a sense as respectful as it was affectionate. After his retirement from active professional life, Dr. HADLEY devoted much of his time to floral and fruit culture, pursuing it with a keen pleasure, engendered by an innate love of the beautiful. A great part of the labor of his little farm-garden was performed by his own hands; and to this out door exercise, to his regular and methodic habits, and to his gentle and equable disposition, his longevity may in some part be ascribed. Dr. HADLEY never spoke unkindly of any one; he was naturally reticent, his words were few and to the point, but always without a sting. During his long and painful illness (enlarged prostate) he never murmured. With his eye on the distant land, steadfast through faith, he was ready and willing to go, but not impatient. He died in his eighty-fifth year. He was born tn Weare, N. H. To the last his tall form was stalwart and unbent—he was like a granite column from his native hills, and like granite in truth, right and integrity, but here the simile ceases; he was guileless as a child, and his heart was gushing with the milk of human kindness. Dr. ROCHESTER then moved the passage of Resolutions appropriate to the occasion.

The committee to whom was assigned the duty of preparing resolutions offered the following, which were unanimously passed:

*Resolved*—That, in the demise of this venerable and highly venerated member of the medical profession, the World of Science has lost an earnest, ardent and most conscientious representative; and the community at large, a citizen who, by his pure, blameless and most exemplary life, exerted an influence for public and private good wherever he had lived or was known.

*Resolved*—That the memory of this able, just and good man is to be deeply cherished, and his unostentatious virtues to be emulated.

*Resolved*—That, while we condole with his family in their bereavement, we unite with them in thanks to Almighty God for the length of days vouchsafed to him, who was truly His servant.

THOS. F. ROCHESTER,
G. F. PRATT,
J. S. TROWBRIDGE,
B. T. WHITNEY.

It was also voted: That the profession attend the funeral in a body, and wear the usual badge of mourning.

JULIUS F. MINER, Secretary.

